# Supplementary material for: Identification of photoperiod-induced specific miRNAs in the adrenal glands of Sunite sheep (Ovis aries)
Source: Front Vet Sci. 2022 Jul 22;9:888207. doi: 10.3389/fvets.2022.888207 (PMC9354845; doi:10.3389/fvets.2022.888207)
Supplement: Supplementary file 1 [file Data_Sheet_1.ZIP › Supplementary file/miRDeep2 file.zip!/miRDeep2 file/Know_Structure/LP42A3/oar-mir-485.pdf]

miRBase precursor : oar-mir-485
Total read count : 1990
oar-miR-485-5p read count : 911
oar-miR-485-3p read count : 1079
remaining reads : 0

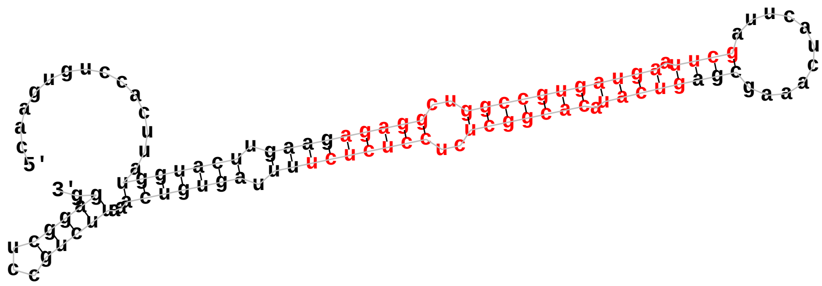

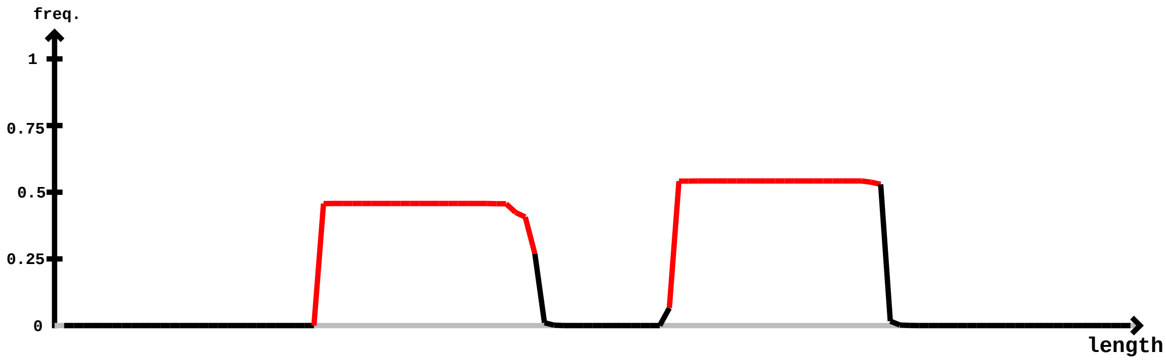


```
                                                     oar-miR-485-5p
                                                                                               oar-miR-485-3p
5'- caaguguccacuuaugguacuugaagagaggcuggccgugaugaauucgauucaucaaagcgagucauacacggcucuccucucuuuuagugucaaauucugccucggagg   -3'    exp
    ((((((.(((....)))))))))((((((((..(((((((((((.((((...........)))))))).)))))))..))))))))...........(((((...))))).    reads     mm      sample
    ..........................agaggcuggccgugauga...................................................................    2         0       seq
    ..........................agaggcuggccgugaugaau.................................................................    65        0       seq
    ..........................agaggcuggccgugaugaauu................................................................    34        0       seq
    ..........................agaggcuggccgugaugaauuc...............................................................    274       0       seq
    ..........................agaggcuggccgugaugaauucg..............................................................    514       0       seq
    ..........................agaggcuggccgugaugaauucga.............................................................    18        0       seq
    ..........................agaggcuggccgugaugaauucgau............................................................    3         0       seq
    ...........................gaggcuggccgugaugaauucg..............................................................    1         0       seq
    ..............................................................agucauacacggcucuccucuc...........................    1         0       seq
    ..............................................................agucauacacggcucuccucucu..........................    128       0       seq
    ..............................................................agucauacacggcucuccucucuu.........................    1         0       seq
    ..............................................................agucauacacggcucuccucucuuu........................    1         0       seq
    ...............................................................gucauacacggcucuccucu............................    9         0       seq
    ...............................................................gucauacacggcucuccucuc...........................    15        0       seq
    ...............................................................gucauacacggcucuccucucu..........................    892       0       seq
    ...............................................................gucauacacggcucuccucucuu.........................    28        0       seq
    ...............................................................gucauacacggcucuccucucuuu........................    2         0       seq
    ...............................................................gucauacacggcucuccucucuuuu.......................    1         0       seq
    .................................................................cauacacggcucuccucucu..........................    1         0       seq
```
